# Associations Between Arterial Stiffness and Executive Function in Older Adults: Preliminary Analysis

**DOI:** 10.1093/geroni/igaf122.3226

**Published:** 2025-12-31

**Authors:** Jeongwoon Kim, Kaetlin Marsh, Kyoung Shin Park

**Affiliations:** Emory University School of Medicine, Atlanta, Georgia, United States; Emory University School of Medicine, Atlanta, Georgia, United States; Emory University School of Medicine, Atlanta, Georgia, United States

## Abstract

Evidence suggests that arterial stiffness is linked to age-related cognitive decline. Majority of research has utilized measures of central tendency (CT) by averaging the outcomes across trials. This approach overlooks intra-individual variability (IIV) which can provide greater sensitivity to subtle cognitive changes. However, the link between arterial stiffness and cognitive IIV remains unexplored. This study utilized preliminary baseline data from ongoing clinical trials to explore how arterial stiffness is associated with IIV and CT scores of executive function. We hypothesized that more arterial stiffness is associated with greater IIV and lower CT scores of executive function. Twelve cognitively unimpaired, female older adults (68.5±2.4 yrs) completed the Flanker and Dimensional Change Card Sort (DCCS) from the NIH Toolbox© V3 to assess cognitive flexibility and inhibitory control, respectively. Arterial stiffness was assessed through carotid-femoral pulse wave velocity (cfPWV) using SphygmoCor XCEL©. IIV outcomes were calculated as the standard deviation of response time (SDRT). CT outcomes utilized the average response time (mRT) across correct trials and the response time corrected accuracy rate (AccRT). Due to limited sample size, the hypothesis was tested using Spearman correlations. For DCCS, cfPWV was associated with greater SDRT (ρ = 0.81, p = 0.001) with a trend level association for mRT (ρ = 0.53, p = 0.075) and AccRT (ρ=-0.55, p = 0.063). cfPWV was not associated with Flanker SDRT, mRT, or AccRT. These findings suggest that IIV offers unique insights into cardiovascular associations with cognitive aging. Furthermore, the selective associations with cognitive flexibility but not inhibitory control suggest that arterial stiffness influences different domains of executive function.

